# Towards a Safe Human–Robot Collaboration Using Information on Human Worker Activity

**DOI:** 10.3390/s23031283

**Published:** 2023-01-22

**Authors:** Luka Orsag, Tomislav Stipancic, Leon Koren

**Affiliations:** Faculty of Mechanical Engineering and Naval Architecture, University of Zagreb, Ivana Lucica 5, 10000 Zagreb, Croatia

**Keywords:** human–robot collaboration, activity recognition, deep learning, LSTM, safe HCI, adaptive manufacturing systems, robotics

## Abstract

Most industrial workplaces involving robots and other apparatus operate behind the fences to remove defects, hazards, or casualties. Recent advancements in machine learning can enable robots to co-operate with human co-workers while retaining safety, flexibility, and robustness. This article focuses on the computation model, which provides a collaborative environment through intuitive and adaptive human–robot interaction (HRI). In essence, one layer of the model can be expressed as a set of useful information utilized by an intelligent agent. Within this construction, a vision-sensing modality can be broken down into multiple layers. The authors propose a human-skeleton-based trainable model for the recognition of spatiotemporal human worker activity using LSTM networks, which can achieve a training accuracy of 91.365%, based on the InHARD dataset. Together with the training results, results related to aspects of the simulation environment and future improvements of the system are discussed. By combining human worker upper body positions with actions, the perceptual potential of the system is increased, and human–robot collaboration becomes context-aware. Based on the acquired information, the intelligent agent gains the ability to adapt its behavior according to its dynamic and stochastic surroundings.

## 1. Introduction

When exploring flexible manufacturing, cyber-physical systems, or Industry 4.0 (I4.0), the term “human–robot collaboration” rarely escapes attention; rather, it encapsulates every aspect of the idea in one self-sustaining concept [1]. Advances in technology increasingly affect our daily activities. We can observe notable progress in AI, leading to the creation of new fields of research, methods, technologies, and their applications, including applications in medicine [2], decision making [3], algorithm development [4], and intelligent agent architecture for security purposes [5].

Recent research and emerging trends can serve as windows that allow us to envisage the future and adopt the concept of collaborating with robotic systems. The concept of industrial manipulators working alongside human co-workers carries the burden of the need to remove barriers and obstacles, allowing us to fully exploit the machines’ strength and dexterity. The main issue and concern with barrierless spaces is the safety of humans and machine operators. Traditionally, it has been the case that industrial robots ignore the occupants of the space and cannot ensure hazardless operation. While methods, such as the use of torque sensors or safety zones can ensure the workers’ safety, they reduce the overall flexibility and efficiency of the system. With recent advancements in machine learning (ML) and computer vision (CV), the field of human–robot collaboration (HRC) is also developing [6]. HRC teams offer a way to use the problem-solving skills of human operators together with robots’ strength and agility. For example, by using a vision-based modality, intelligent agent can acquire additional high-level information about its surroundings and, in the context of safety, information about the human co-workers. Such information can be presented as motion information (poses, joint velocities, etc.) and even human emotional states, which can be represented through facial expressions or body language [7]. While this set of new information is open to interpretation through careful consideration, it can form contexts and minimize the stochastic factors present in collaborative industrial environments.

HRC is a relatively young scientific field, in which researchers are developing control models and other means to render this collaboration safe and efficient [8,9,10]. The methods that enable us to achieve these goals can be designed from both physical and cognitive perspectives [11]. At the physical level, researchers are designing and building collaborative robots (cobots) that can be used to share the same physical interactive space with humans [12]. Such robots are usually in close proximity with humans while performing joint work tasks. The collaborative robot or cobot can engage in safe interaction, based on special software and smart sensors that are ubiquitously placed within the interaction space. The physical components of such robots usually have rounded edges and are constructed from lightweight construction materials. During the interaction, the robot parts are moved carefully, with limitations on their speed and force [13].

The design of HRC, at the cognitive level, presumes the use of input sensor information about the environment in which the user is positioned [14]. This information is then elaborated and translated to the robot, in which a computation model designed to drive the robot’s behavior shapes the robot’s responses. A thorough overview on the design of learning strategies for HRC is provided in [15]. These strategies are based on ML, which is a promising field, especially with the rise of deep learning (DL), which is often used to train computation models, based on data about different domains of interest in the form of case knowledge.

Based on their applications, different ML techniques can be employed to build HRC models. According to action (activity) and intention recognition applications, which are the focus of this work, there are also a couple of interesting approaches that rely on different sensing modalities. It is, therefore, important to exploit the value of each modality for the purpose of better action recognition [16]. For example, a vision-based action analysis can focus on different characteristics of the input data, such as the RGB, depth, skeleton, and infrared (IR) [17]. Signal segmentation is also an important stage in the action recognition process [18]. In [19], the authors proposed a method that can be employed to achieve a fast and fluid human–robot interaction by estimating the progress of the human co-workers’ movements.

In recent years, researchers have begun to collect data that could be used to build DL models with action recognition HRC applications. These data are often difficult to analyze and annotate [20,21,22]. For example, in [23], the authors distilled spatial and temporal data representing human actions using convolutional neural networks (CNN) and long short-term memory (LSTM). In [24], an approach based on transfer learning is proposed, thus avoiding the need for a large amount of annotated data, whose collection is often a tedious job to perform. In [25], activity recognition based on built-in sensors in smart and wearable devices and custom-made HHAR-net is investigated.

Intention recognition is a more complex task, compared to the recognition of actions. It represents the task of recognizing the intentions of a person by analyzing his/her actions and/or changes in the environment caused by these actions. Intention-based approaches to human–robot interaction is discussed in [26]. The social component is also an important aspect of worker–robot interaction [27]. The way in which a person behaves during the interaction can be analyzed and employed for the purpose of intention recognition. For example, the movements of the worker’s body can be associated with a possible collision with the robot [28]. In [29], the authors proposed an addition to a safety framework using a worker intention recognition method, based on head pose information. In [30], the authors described a behavior recognition system, based on a real-time stereo face-tracking and gaze detection designed to measure the head pose and gaze direction simultaneously. Additionally, a fusion technique for the improvement of intention recognition performance was proposed in [31], and different information sources were explored in [32].

In [33], an intention-guided control strategy was proposed and applied to an upper-limb power-assisted exoskeleton. In [34], the authors proposed a systematic user experience (UX) evaluation framework of action and intention recognition for interactions between humans and robots, from a UX perspective. In [35], the authors presented a method based on the radial basis function neural network (RBFNN) to identify the motion intention of the collaborator.

This work aims to develop efficient HRC in industrial settings. Sensors have a significant impact in the modeling of context-aware industrial applications in which vision plays a special role, as emphasized in [36,37]. Context awareness synchronized with significant environmental occurrences is key to improving operational efficiency and safety in HRC for the purpose of intelligent manufacturing [38,39]. Context-aware systems enable a transition towards more flexible production systems that are rapidly adjustable, in response to changing production requirements [40]. In [41], the authors proposed a hybrid assembly cell for HRC that supervises human behavior to predict the demand for collaborative tasks. There are other applications based on similar principles and methodologies. For example, in [42], the authors quantified the uncertainties involved in the assembly process by constructing a model of mutual trust between humans and robots.

The approach presented in this work relies on the analysis of micro- and macro-movements between interacting units within an industrial environment. Joints connected with links are the natural parts of a unit’s skeleton, representing a moving environmental structure. The skeleton can represent a person, robot, or any other natural or artificial mechanism. The micro-movements of the skeleton usually have a contextual meaning and different consequences within the environment. An analysis of this cause–effect relationship within a temporal and spatial continuum is provided here to empower the intention and activity recognition model. In the case of the model presented herein, generic movements (e.g., picking left, picking in front, etc.), as opposed to the specific actions that the worker can perform (e.g., using a screwdriver, applying grease, etc.), are evaluated and used. Consequently, the model is expected to support the development of skills for system adaptation in response to constant environmental changes. We propose a development framework for the planning of safe robot actions using human worker poses, estimated by a neural network (NN) module dedicated to the prediction of human joint locations. In this way, the robotic manipulator is provided with the information required to ensure human safety and maintain efficiency. The activity recognition module provides the contextual information required for pose extrapolation, thus decreasing the system’s reaction time, similar to the work described in [43,44]. The difference between these two approaches is the information input to the AR module and the selection of the output classes. In [43], the authors proposed a task-oriented action recognition system which relies on the topology of the worker station, as opposed to generic human worker actions. We believe that from the perspectives of safety and system flexibility, activities should be generalized and inherited by different robotic cell topologies.

**Figure 1:**
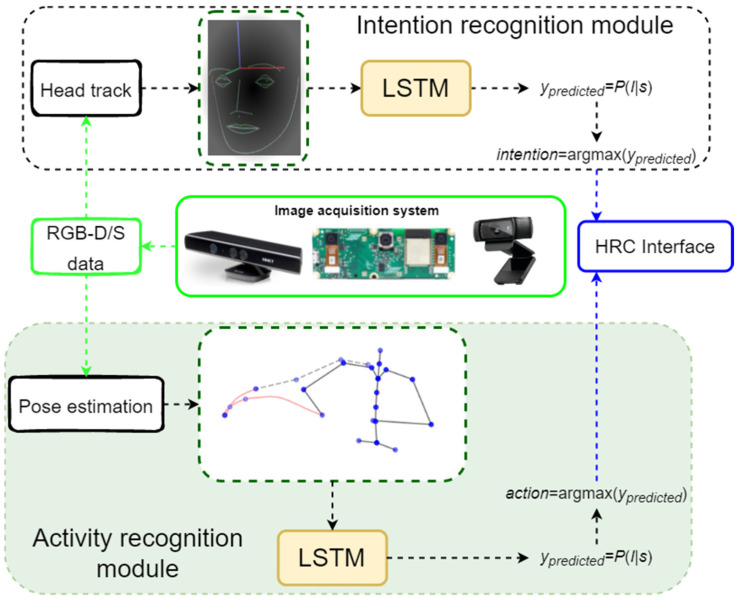
Safe HRC framework, based on human activity recognition and pose prediction.

The activity recognition (AR) module shown in Figure 1 (highlighted with a green background) is modelled using long short-term memory (LSTM) networks and consists of a classification module for AR.

The remainder of the paper is organized as follows:

- Section 2: Through an overview, the model is introduced, and certain pose estimation models are described and explained. We present an overview of the dataset used for the activity recognition. Furthermore, the algorithms and explanations that summarize the work performed for the purpose of action recognition are discussed, and finally, the overall framework of the system’s simulation and visualization is explained;
- Section 3: In this section, the training and overall results of the system are discussed;
- Section 4: In the final section of this paper, we summarize the work conducted and propose future directions for the field.

## 2. Materials and Methods

The proposed model is based on the action recognition module (ARM). It employs deep learning techniques rather than traditional ones, since they have a proven capacity to perform well without feature selection and engineering. Together with the obvious reasons, there is a lack of understanding about how the basic actions are performed in stochastic environments. For example, the action of “*Picking Left*” is an action that is performed by using either the left or right hand, which renders the features difficult to analyze when expressed as multi-variate time-series.

### 2.1. Pose Estimation

Human pose estimation (HPE) is a way of identifying and classifying the joints in the human body. In essence, it is a method used to capture a set of coordinates for each joint, known as a key point, that can describe a pose. With the key points described, not all of them can form a pair. Association techniques are employed to form a skeleton-like representation of the human body.

In this section, we briefly reflect on a possible human pose estimation method for obtaining the skeleton view for the purpose of this work. It is possible to identify three main types of human pose estimation models that can be used to represent the human body in 2D and 3D spaces (skeleton-based, planar-based, and volumetric). For this work, a skeleton-based is preferred, since the relationships between the joints are used to represent the activities performed by the human worker in a collaborative environment.

### 2.2. Dataset

The InHARD dataset is a large-scale RGB + skeleton action recognition dataset named the “Industrial Human Action Recognition Dataset” [21]. It includes 4804 different action samples represented in over 38 videos collected from 14 industrial action classes. In comparison with other existing action recognition datasets which comprise daily activities, the authors of [21] proposed a method, based on actual industrial actions performed in real use-case scenarios in an industrial environment. Together with the dataset, usage metrics are proposed for the purpose of algorithm evaluation. The RGB data are recorded from three different angles (top, left side, and right side) to capture the complete action and help to improve the ML algorithm’s performance in cases where occlusion occurs.

**Figure 2:**
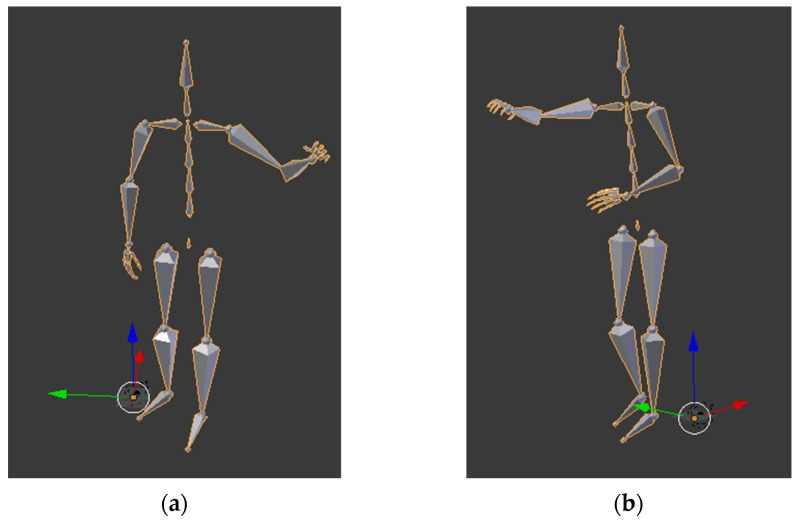
InHARD skeleton preview in Blender: (**a**) picking left (**b**), picking in front.

For the *skeleton* modality, a *Combination Perception Neuron 32 Edition v2* motion sensor was used to capture MOCAP data at a frequency of 120 Hz [21]. The skeleton data comprises the 3D locations (*Tx*, *Ty* and *Tz*) of 17 major body joints, together with their rotations (Rx, Ry, and Rz). The data are saved in standard BVH file format and can be examined using various software packages, e.g., the Blender software, as shown in Figure 2.

**Figure 3:**
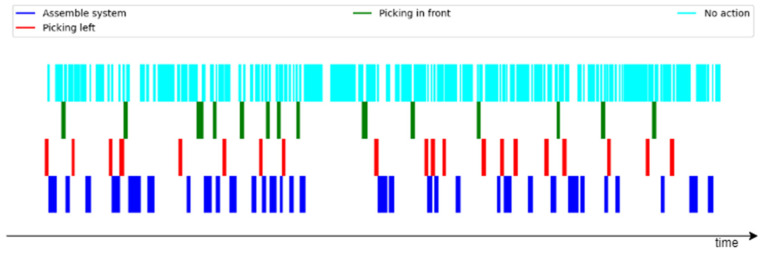
Relevant activity event distribution over one recording in the InHARD dataset.

The authors of [21] identified 14 different low-level classes, as presented in Table 1, and 72 high-level classes, in which the actions are much more accurate. For the purpose of this experiment, a subset of 4 low-level classes were used. A visual representation of the time distribution of these four classes during one recording is presented in Figure 3.

### 2.3. Dimensionality Reduction

**Figure 4:**
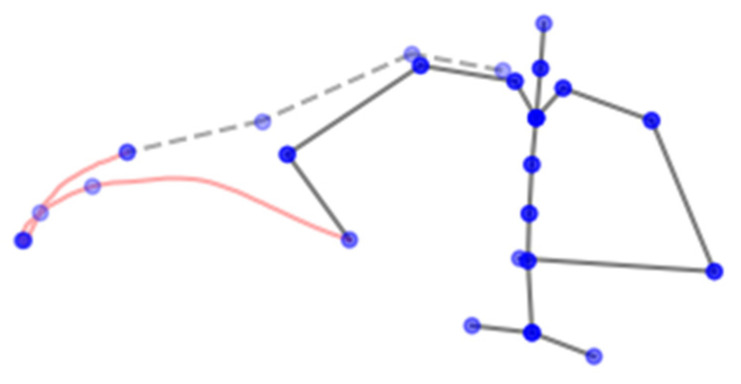
Skeleton hierarchy considered as the input to LSTM.

Upon the dataset’s analysis, it was determined that not all of the feature points of the skeleton hierarchy are necessary, and some can be excluded from the training of the model. The new skeleton hierarchy comprises points that are visible above the assembly surface, since the features are not visible to the camera in that area and deemed unnecessary for the purpose of activity recognition. Figure 4 represents a new hierarchy and an example of a relevant extremity trajectory.

A subset of joints *J* are then defined as an array with a length of *k* (17, in this case), resulting in $J={\left\{j_{i}\right\}}_{i=1}^{k}$.

**Figure 5:**
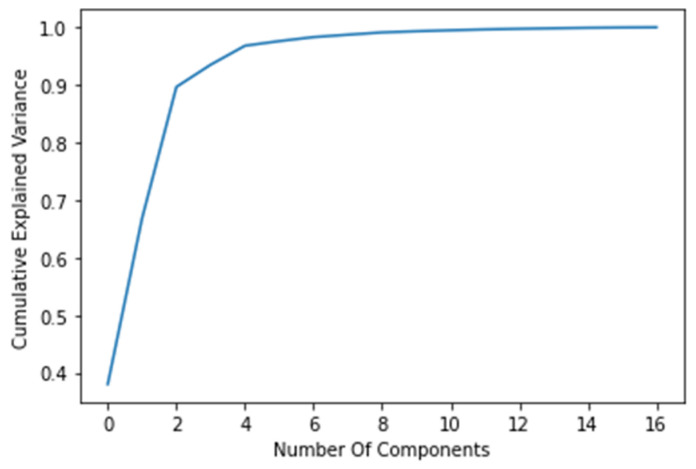
Cumulative explained variance ratio as a result of the PCA.

Subset *J* can be reduced further by applying a principial component analysis (PCA) to the remaining dataset. The results are presented in Figure 5, and the dataset is further reduced (9 joints, in this case, resulting in 27 input features).

### 2.4. Human Activity Recognition

HAR aims to understand human behaviors, which enable the computing systems to proactively assist users, based on their requirements [45]. From a formal perspective, suppose that a user is performing activities belonging to a predefined activity set *A*:

(1) $$A={\left\{a_{i}\right\}}_{i=1}^{m},$$

where *m* denotes the number of activity classes. There is a sequence of sensor readings that capture the activity information:

(2) $$s=\left\{d_{1},\;d_{2},\cdots ,d_{t},\cdots ,d_{n}\right\},$$

where $d_{t}$ denotes the sensor reading at time *t*. We must build a model F to predict the activity sequence, based on sensor reading *s*:

(3) $$\hat{A}={\left\{{\hat{a}}_{j}\right\}}_{j=1}^{n}=F\left(s\right),\;\;\;\;{\hat{a}}_{j}\in A,$$

while the true activity sequence (ground truth) is denoted as:

(4) $$A^{*}={\left\{a_{j}^{*}\right\}}_{j=1}^{n},\;\;\;\;a_{j}^{*}\in A$$

given that n≥m. We then select a positive loss function $L\left(F\left(s\right),A^{*}\right)$ to minimize the discrepancy between $\hat{A}$ and $A^{*}$. In this work, a multi-class categorical cross-entropy loss function is used:

(5) $$L\left(F\left(s\right),A^{*}\right)=-\overset{n}{\underset{c=1}{\sum }}a_{c}^{*}\log\left(P\left({\hat{a}}_{c}\right)\right)$$

The list of output classes comprises four actions acquired from the InHARD dataset, forming an output vector *A*. The actions include those highlighted in Table 1.

Every joint can be expressed as a point $P_{J_{k}}=\left\{X_{J_{k}},Y_{J_{k}},Z_{J_{k}}\right\}$ in a cartesian coordinate system, indexed in chronological order. A set of such joints can be used to describe the actions and can be analyzed by a neural network in fixed timeframes. In this case, one sensor reading $d_{t}$ appears as:

(6) $$d_{t}=\left\{P_{j_{1}},P_{j_{2}},\cdots ,P_{j_{k}}\right\},$$

**Figure 6:**
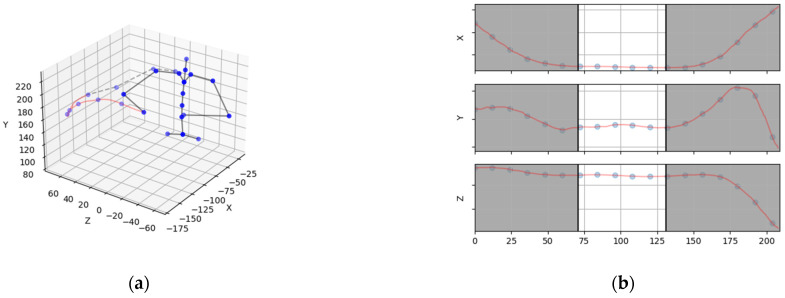
Image representing the skeleton data used for training the NN model and the sliding window approach: (**a**) view of the skeleton data with the points and motion data for one point (a hand motion is presented in this example); (**b**) hand motion presented as timeseries.

Vector s in (2) is described as a sequence of sensor readings. It is defined as a sliding window and can be used for human activity analysis using complete sets of data, where $d_{n}$ is a moment surpassing $d_{t}$, representing a future joint movement. In HRC environments, sensors can obtain only present or past sets of events. A sequence of sensor readings are then expressed as:

(7) $$s=\left\{d_{t-n},\cdots ,d_{t-2},d_{t-1},d_{t}\right\},$$

and transposing (6) and placing it into (7) gives:

(8) $$s=\left\{{\left[\begin{array}{c} P_{j_{1}}\\ P_{j_{2}}\\ \begin{array}{c} \vdots \\ P_{j_{k}} \end{array} \end{array}\right]}_{t-n},\cdots ,{\left[\begin{array}{c} P_{j_{1}}\\ P_{j_{2}}\\ \begin{array}{c} \vdots \\ P_{j_{k}} \end{array} \end{array}\right]}_{t-2},{\left[\begin{array}{c} P_{j_{1}}\\ P_{j_{2}}\\ \begin{array}{c} \vdots \\ P_{j_{k}} \end{array} \end{array}\right]}_{t-1},{\left[\begin{array}{c} P_{j_{1}}\\ P_{j_{2}}\\ \begin{array}{c} \vdots \\ P_{j_{k}} \end{array} \end{array}\right]}_{t}\right\},$$

and it is visualized in Figure 6.

**Figure 7:**
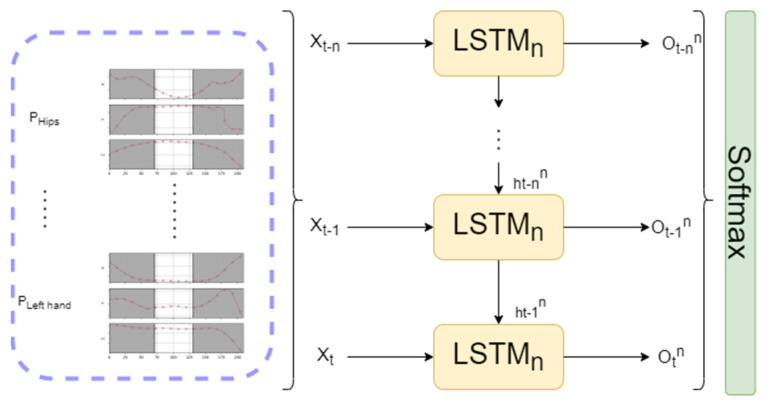
Overview of the complete LSTM NN for activity recognition.

The selected model implements DNN with hidden LSTM layers (Figure 7). We used the rectified linear activation function (ReLu), since it overcomes the vanishing gradient problems present in RNNs [46,47]. It also allows models to learn faster and perform better. The Softmax function is used as an output layer from NN, since the desired output is a vector of probabilities. The probabilities of each value are proportional to the relative scale of each value in the vector and are interpreted as probabilities of the membership of each class.

The probabilities of each value are proportional to the relative scale of each value in the vector and are interpreted as probabilities of the membership of each class. The ${\hat{a}}_{class}$ takes the form of:

(9) $${\hat{a}}_{class}=P\left(a|s\right)$$

and the final output is calculated as follows:

(10) $$\hat{y}=argmax\left(\hat{A}\right)$$

The NN module is implemented with the help of Keras API, which is an open source NN library written in Python [48]. The trainings are performed in 500 epochs with a batch size of 64.

### 2.5. Simulation and Visualization Environment

**Figure 8:**
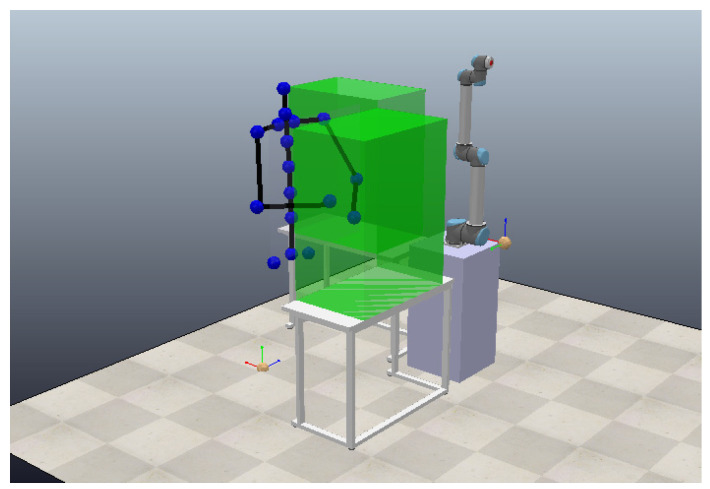
HRC team modelled using the CoppeliaSimEdu software package.

In this study, the HRC environment was modeled and implemented using the CoppeliaSimEdu software package [49]. CoppeliaSim is a robotic environment simulator with an integrated development environment. It is based on distributed control architecture, meaning that each object can be individually controlled via an embedded script, a plugin, ROS node, remote API client, or a custom solution. In this work, the HRC environment was modeled as a workspace shared by human and robot partners forming a manufacturing team. The workspace consists of two tables representing work surfaces with a robot on a mount and a space for the human worker, as presented in Figure 8. The human worker is represented as a set of joints acquired from the InHARD dataset. Green boxes are placed on top of the work surfaces to represent the event-trigger-activated safety zones.

**Figure 9:**
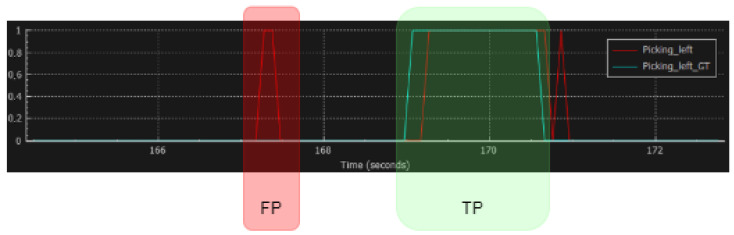
An example of an implemented CoppeliaSimEdu *Graph* element.

In addition to a visual worker representation, CoppeliaSimEdu’s *Graph* element is deployed to represent the actions detected by the neural network. An example of an implemented graph element is presented in Figure 9.

The example presented in Figure 9 demonstrates the distinction between the *True Positive* and *False Positive* detections (marked as *TP* and *FP* in the image). Here, we used the graph for the visual inspection and the arbitrary analysis of the proposed model’s performance. We found this method useful, since the detections are based on a temporal component, and the *TP* detection can be described as a set of predictions generated by the model that sufficiently overlaps with the ground truth (annotations) on a temporal axis of the graph. The *FP* detection can be described in the same manner as a model-generated prediction without sufficient overlap with the ground truth on a temporal axis. For the purpose of this work, sufficient overlap was determined to be arbitrary.

## 3. Results and Discussions

In this section, we present the training and simulation results. Following the training of the AR network, the accuracy and confusion matrices were generated. We also discuss the results of the PCA and dimensionality reduction, together with the overall results, which were evaluated through a visual inspection in the CoppeliaSimEdu environment.

### 3.1. Dimensionality Reduction Results

The results of the dimensionality reduction by the PCA are displayed in Table 2. The results are displayed as the number of feature points, which are represented as *X*, *Y*, and *Z* components of the human skeleton joints. The total number of joints can be calculated as the resulting number of feature points divided by three.

### 3.2. Action Recognition Model Training Results

**Figure 10:**
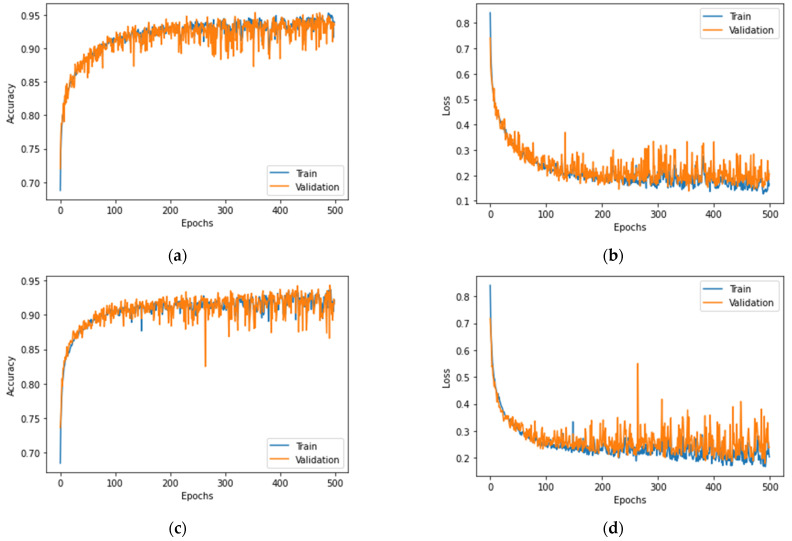
Accuracy and loss plots for training and validation: (**a**) accuracy plot for the network with 51 input features; (**b**) training and validation loss for the network with 51 input features; (**c**) accuracy plot for the network with 27 input features; (**d**) training and validation loss for the network with 27 input features.

Two networks with five hidden layers were trained: (1) a network with 51 input features, and (2) a network with 27 input features. The networks were trained in 500 epochs with batch sizes of 64. The neural network with 51 input features scored ~93% for accuracy, based on the validation data, while the network with 27 input features scored 91.365% for accuracy. Figure 10 provides insight into the training results, from which we can draw several conclusions. The accuracy plots in Figure 10a,c provide information about the convergence, accuracy discrepancy, and usability of the dimensionality reduction methods employed in this case. Both networks can achieve a good accuracy with a small discrepancy of ~1.7%, which is acceptable in this case. The training and validation losses for both networks, displayed in Figure 10b,d, converge well before the 500th epoch, suggesting a lack of overfitting or underfitting.

**Figure 11:**
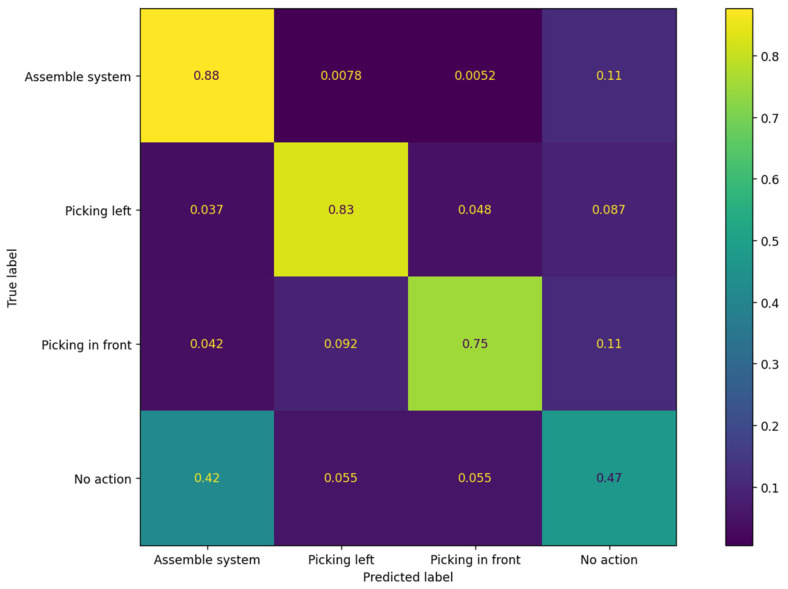
Confusion matrix for the model with 27 input features (nine joints).

Since the accuracy alone is not enough to evaluate the models’ performance, the precision, recall, and F1-score were also calculated (Table 3). The confusion matrix for one of the models is presented in Figure 11.

The values presented above and the F1-score analysis show that the two models have a similar performance. To explain the lower F1-score value, the confusion matrix can be consulted, as shown in (Figure 11), from which a few conclusions can be obtained:

- The dataset appears to be unbalanced. The *Assemble System* action comprises most of the dataset;
- Actions, such as *Assemble System* and *No Action,* are very similar in terms of motion and show little variance;
- A large portion of every action class is predicted as *No Action*. The explanation for this trend lies in the fact that the beginning and end of each action starts with the same motion properties.

The results of the confusion matrix can be compared to the results of similar work described in [43]. The authors presented their results using four confusion matrices for each model, based on the motion completion percentage (25, 50, 75, and 100% completion). We compared the results regarding the 25% and 50% completed motions. Since the output classes of the network are different, we selected the *Picking In Front* and *Picking Left* classes as references, as these are most similar to the motions discussed in [43]. The authors of [43] were faced with similar issues when evaluating their similar classes for 25% motion completion. We encountered such issues in the case of the *No Action* and *Assemble System* classes, where 42% of the *No Action* class is recognized as the *Assemble System*.

### 3.3. Online Performance Results

Together with the training results, we explored the online performance of the AR model via visual inspection using *CoppeliaSimEdu* graphs and validated the conclusions, based on the confusion matrix. As presented in [21], *online recognition* is defined as the detection of the on-the-fly recognition within a long video sequence, performed as early as possible without using any further information. The authors of [21] (see the section on ***online metrics***) also explored the *online* performance accuracy, calculated for each class that we defined in the NN output. Table 4 depicts the results regarding the performance of the *online* model.

**Figure 12:**
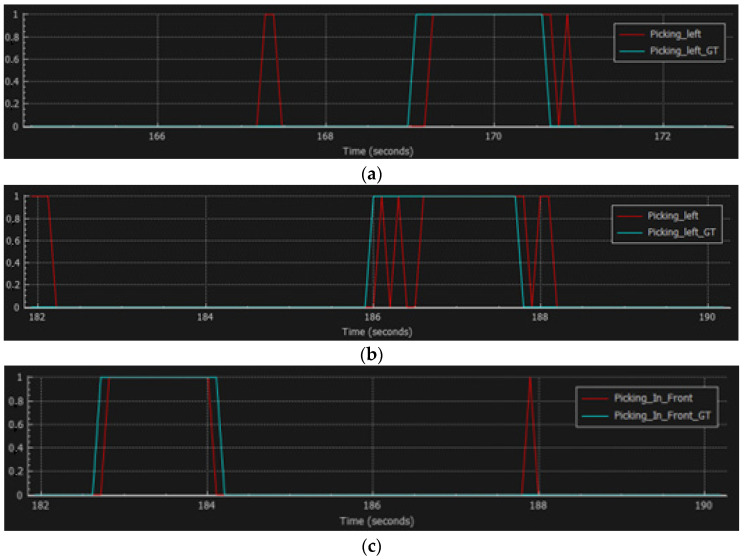

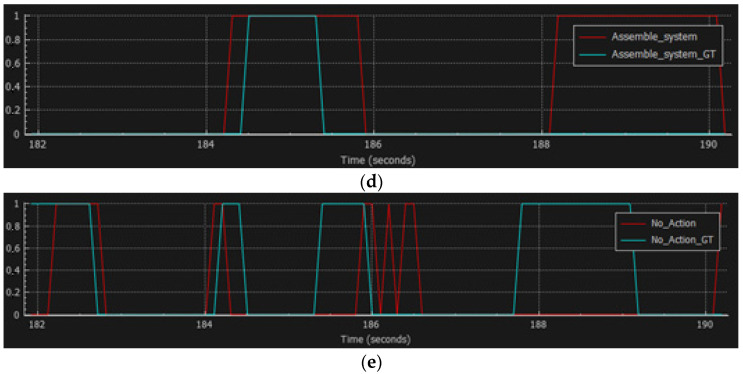
Online model performance results: (**a**) event plot for the *Picking Left* class; (**b**) event plot for the *Picking Left* class (second example); (**c**) event plot for the *Picking In Front* class; (**d**) event plot for the *Assemble System* class; (**e**) event plot for the *No Action* class.

Figure 12 provides examples of event plots that explain this behavior of the model. The graph plots visualize the model predictions and the ground truth in the time intervals in which they occur.

In Figure 12a–c, the classes of *Picking Left* and *Picking In Front* are evaluated. A visual inspection shows that the classifier performs well while working *online* (the entire sequence is processed) regardless of the lower accuracy. In this example, the ground truth interval starts at the ~169th second and ends at the ~170.7th second, while the predictions are shifted by ~0.2 s. In this example, some false positives can be observed, but they are short-lasting and can be mitigated by further processing. A similar performance is observed for the action of *Picking in Front*. As the acceptance criterion is set to 60% of the ground truth coverage for each detection interval, the reason for the lower performance, compared to that of the confusion matrix in Figure 11 becomes clear.

When evaluating the classes, such as the *Assemble System* and *No Action* (Figure 12d,e), noticeable model confusion can be observed. In this example, the ground truth of the *No Action* class in Figure 12e starts at the ~187.8th second and ends at the 189.2nd second. The *Assemble System* class overlaps with this time interval instead of the *No Action* class. Similar to the example shown in Figure 12a, false positives can be observed, together with the time shift of the detections. In this case, the time shift is more noticeable, as the detections do not provide the true positive detection.

Together with the notable false positives for the *No Action* and *Assemble System* classes, intermittent detections can also be observed for both classes.

## 4. Conclusions and Future Work

In this work, a skeleton-based trainable model was developed to classify human worker actions in an industrial environment. The model uses LSTM hidden layers for the purpose of spatiotemporal activity classification, based on an approach that combines the human worker positions and actions to increase the perceptual potential of the system. In this way, human–robot collaboration becomes context-aware, as the model provides information to the intelligent agent, enabling it to adapt its behavior, based on the changes in its dynamic and stochastic surroundings.

Machine-vision-based skeleton pose estimation provides a useful set of information for the purpose of efficient human worker activity recognition. By breaking the pose down into its components (joints), spatiotemporal information can be extracted, providing the input for the AR model.

The AR model comprises five hidden LSTM layers used for activity classification. Here, four classes were evaluated and represented as an output of the AR model. The model evaluation techniques show that the system successfully recognizes the activities of the human worker. While the accuracy indicates the acceptable performance of the model, the confusion matrix reveals that there is still room for improvement, as some of the classes are recognized as false positives, especially in the case of the *Assemble System* and *No Action* classes. False positives themselves do not represent a critical problem; rather, their intermittent behavior during short time intervals are problematic, since they can be regarded as true positive detections. In the future, these problems will be mitigated by post-processing and filtering, together with a multimodal information fusion, based on the recognition of human worker intention, as shown in [29].

The work presented in this paper, together with the work discussed in [29], represents the building blocks for efficient HRC, the final goal of this research, in which the robot and the worker can closely cooperate.

## Figures and Tables

**Table 1:** InHARD dataset low-level action classes. The highlighted classes are chosen as the action classes of interest.

Action ID	Meta-Action Label
0	**No action ^1^**
1	Consult sheets
2	Turn sheets
3	Take screwdriver
4	Put down screwdriver
5	**Pick in front ^1^**
6	**Pick left ^1^**
7	Take measuring rod
8	Put down measuring rod
9	Take component
10	Put down component
11	**Assemble system ^1^**
12	Take subsystem
13	Put down subsystem

^1^ Actions considered for the purposes of this article.

**Table 2:** Overview of the dimensionality reduction results.

Dimensionality Reduction Method	Resulting Number of Feature Points	Comment
None ^1^	63	N/A
Visual inspection ^2^	51	N/A
PCA	27	Human worker symmetry is compromised ^3^

^1^ No dimensionality reduction method is performed, and the dataset remains the same. ^2^ An engineering assumption is made, according to which the lower part of the body (up to the worker’s hips) will be omitted in the setup, as presented in Figure 8. ^3^ One of the joints is deemed unnecessary for the dataset’s description due to the low *explained variance ratio* value. Following the PCA performance, the dataset is analyzed to confirm the validity of the results. The *LeftArm* joint is in a position where it does not need to be moved to a large extent.

**Table 3:** Precision, recall, and F1-score for the trained models.

Metric	Model with 51 Input Features	Model with 27 Input Features
Precision	0.709	0.709
Recall	0.699	0.694
F1-Score	0.686	0.680

**Table 4:** Online accuracy results for each subclass.

Subclass	Accuracy (%)
*Picking In Front*	~63%
*Picking Left*	~67%
*No Action*	~39%
*Assemble System*	~38%

## Data Availability

No new data were created or analyzed in this study. Data sharing is not applicable to this article.
